# *Emergomyces canadensis,* a Dimorphic Fungus Causing Fatal Systemic Human Disease in North America

**DOI:** 10.3201/eid2404.171765

**Published:** 2018-04

**Authors:** Ilan S. Schwartz, Stephen Sanche, Nathan P. Wiederhold, Thomas F. Patterson, Lynne Sigler

**Affiliations:** Global Health Institute, University of Antwerp, Antwerp, Belgium (I.S. Schwartz);; San Antonio Center for Medical Mycology, UT Health San Antonio, San Antonio, Texas, USA (I.S. Schwartz, N.P. Wiederhold, T.F. Patterson);; University of Saskatchewan, Saskatoon, Saskatchewan, Canada (S. Sanche);; UT Health San Antonio Fungus Testing Laboratory, San Antonio (N.P. Wiederhold);; South Texas Veterans Health Care System, San Antonio (T.F. Patterson);; University of Alberta Biological Sciences, Edmonton, Alberta, Canada (L. Sigler)

**Keywords:** Emergomyces canadensis, Es. canadensis, Onygenales, Emmonsia, emergomycosis, opportunistic infections, mycology, mycoses, invasive fungal infections, dimorphic, fungi, North America

## Abstract

We report 4 patients in North America with disease caused by *Emergomyces canadensis,* a newly proposed species of pathogenic dimorphic fungus. Affected persons were immunocompromised; lived in Saskatchewan, Colorado, and New Mexico; and had systemic disease involving blood, skin, cervix, lung, and lymph node. Two cases were fatal.

Members of the recently described fungal genus *Emergomyces* cause disseminated and often fatal disease in immunocompromised hosts (*1*,*2*). So named because of their recent global emergence (*1*), these dimorphic fungal pathogens have been reported from Africa, Asia, and Europe (*3*). Here we report from North America 4 cases of invasive fungal disease caused by a novel *Emergomyces* species, designated *Es. canadensis*.

## The Study

In 2003, a 39-year-old man with a history of diabetes and a cadaveric renal transplantation 3 years prior visited a hospital in Saskatoon, Saskatchewan, Canada, reporting fever and throat pain. His medications included mycophenolate and prednisone (25 mg/d). The patient had no history of travel. He kept pet birds, none of which were ill, and had no other animal exposures.

On examination, the patient was cushingoid, normotensive, and afebrile. Results of oropharyngeal, chest, and abdominal examinations were unremarkable. Chest radiograph and computed tomography demonstrated diffuse micronodules, left upper lobe consolidation, and mediastinal lymphadenopathy. The patient was assessed by esophagoscopy, which indicated white, dry patches suspicious for esophageal candidiasis; consequently, we started him on oral fluconazole (200 mg/d). On day 5 postadmission, he had myalgias, arthralgias, and a fever of 38.9°C, prompting collection of mycobacterial and fungal blood cultures. Two days later, a bronchoscopy demonstrated white patches in the trachea and bronchi. On day 17 of admission, both the blood and bronchoalveolar lavage cultures grew a fungus. Repeated blood cultures subsequently grew the same fungus. We treated the patient with lipid complex amphotericin B for 3 weeks, with clinical improvement, and he was discharged after 7 weeks in the hospital.

**Figure 1:**
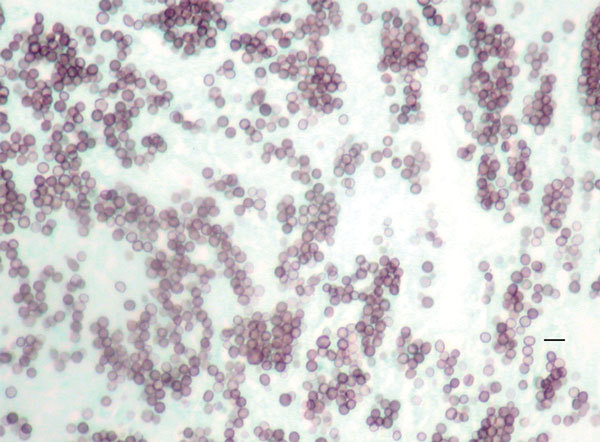
Methenamine silver stain of mediastinal lymph node biopsy, demonstrating small round or oval yeasts in tissue, from a patient infected with novel fungal species *Emergomyces canadensis* (case-patient 2), Saskatoon, Saskatchewan, Canada, 2003. Scale bar indicates 10 µm.

Several days later, the patient returned, reporting weakness, postural dizziness, anorexia, and vomiting. Repeated chest radiograph and computed tomography showed patchy consolidation with increased right mediastinal lymphadenopathy. A mediastinoscopy with lymph node biopsy excluded lymphoma. No material was sent for culture, but histopathologic examination with fungal stains demonstrated small, round or oval yeasts (Figure 1). The patient remained afebrile, and he was managed expectantly without additional antifungal therapy. His symptoms resolved, and he was discharged. Serial chest radiographs demonstrated resolution of the mediastinal lymphadenopathy, and no clinical relapse occurred in 3 years of follow-up.

**Figure 2:**
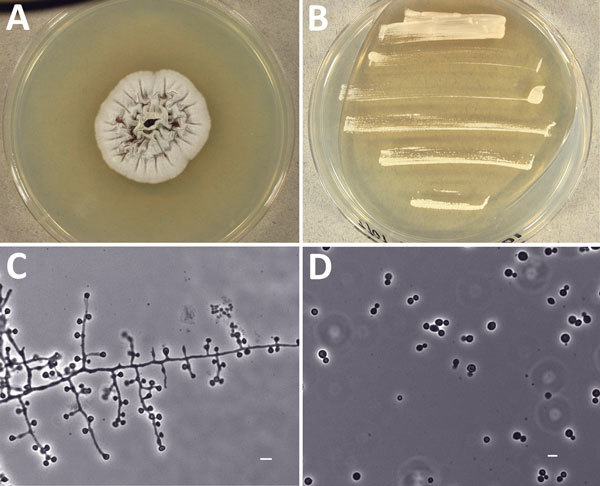
Morphologic features of novel fungal species *Emergomyces canadensis* isolated from case-patient 2, Saskatoon, Saskatchewan, Canada, 2003. A–B) Colonies grown on potato dextrose agar showing mold phase after 28 days at 30°C (A) and yeast phase after 9 days at 35°C (B). C) Mycelial phase showing 1–3 conidia borne at the ends of slightly swollen conidiophores or sessile on hyphae. D) Round to oval yeast cells with narrow-based budding produced at 35°C. Scale bars indicate 5 *µ*m.

The clinical isolate was referred to the University of Alberta Microfungus Collection and Herbarium (UAMH) for characterization and identification. The fungus grew as a mold phase at 30°C and as a yeast at 35°C (Figure 2, panels A and B). Microscopic examination of mycelia demonstrated florets of 1 to 3 conidia borne at the ends of slightly swollen conidiophores (Figure 2, panel C), reminiscent of *Emmonsia*-like fungi (*1*,*2*). The yeast cells were small (2.5–5.0 µm) and round or oval, with 1 or occasionally 2 narrow-based buds (Figure 2, panel D).

DNA genetic analysis was performed by sequencing the internal transcribed spacer region and D1/D2 domain of the large subunit rDNA as previously described (*4*). The sequence of the case isolate was identical to one from another clinical isolate obtained in 1992 from another patient in Saskatchewan and thought to represent an undescribed *Emmonsia* species (*4*,*5*). Recent phenotypic and phylogenetic analyses confirmed that these isolates represent a new species within the genus *Emergomyces* (*2*); the proposed name is *Es. canadensis* (Y. Jiang, S. de Hoog, pers. comm., 2018 Jan 19).

We searched for additional clinical isolates among those referred to the Fungus Testing Laboratory at UT Health San Antonio (San Antonio, TX, USA) during 2001–2016. We reviewed isolates previously identified as *Emmonsia* species or as *Blastomyces dermatitidis* based on low-positive (<400,000 relative light units) results on a *B. dermatitidis* DNA probe (AccuProbe, Hologic, Inc., San Diego, CA, USA). We confirmed identification by sequencing of the internal transcribed spacer and D1/D2 regions and thus identified 2 additional isolates of *Es. canadensis*.

We compiled and summarized epidemiologic and clinical data from submitting laboratories and clinicians for the 4 clinical *Es. canadensis* isolates (Table). Two isolates were referred from Saskatchewan, 1 from Colorado, and 1 from New Mexico. All 3 patients for whom medical histories could be ascertained were immunocompromised, 2 with advanced HIV infection and the third with a kidney transplantation. Histopathology results were available for 2 patients: in case 2, small yeasts were observed in tissue from mediastinal lymph node, and in case 3, in tissue from an endocervical mass. *Es. canadensis* was cultured from biopsied cutaneous lesions in 2 patients (in cases 1 and 4). For 2 remaining patients, *Es. canadensis* was cultured from blood, and additionally in 1 patient, from bronchoalveolar fluid. Two patients survived, and 2 died.

**Table:** Epidemiologic and clinical characteristics of 4 patients infected with *Emergomyces canadensis*, North America, 1992–2015

Case no.	Year	Location	Patient age, y/sex	Medical history	Clinical syndrome	Specimen cultured	Treatment	Outcome	Strain ID†
1	1992	Regina, Saskatchewan	68/M	HIV	Sepsis, skin lesions	Skin biopsy	–	Died	UAMH 7172 (AF038322)
2	2003	Saskatoon, Saskatchewan	39/M	Kidney transplant, diabetes	Pneumonia, tracheitis, lymphadenopathy, esophagitis, sepsis	Blood, BAL fluid	Fluconazole, then amphotericin B	Survived	UAMH 10370 (EF592151)
3	2010	Colorado Springs, Colorado	75/F	–	Fungemia, endocervical lesion	Blood	–	Died	UTHSCSA DI17–85 (MG777526, MG777527)
4	2015	Santa Fe, New Mexico	40/M	HIV	Pneumonia, skin lesions, sepsis	Skin biopsy	–	Survived	UTHSCSA DI17–84 (MG777530, MG777528)


*BAL, bronchoalveolar; ID, identification; UAMH, UAMH Centre for Global Microfungal Biodiversity; UTHSCSA, University of Texas Health Science Center at San Antonio; –, data missing. †GenBank accession numbers for relevant nucleotide sequences are given.

We performed limited antifungal susceptibility testing for 2 isolates (*6*). The MIC of UAMH 10370 was 0.125 µg/mL for amphotericin B. The MIC of UTHSCSA DI17–85 was 64 µg/mL for fluconazole and 0.125 µg/mL for itraconazole.

## Conclusions

*Es. canadensis* is one of several newly recognized species within *Emergomyces* (*2*), and causes an endemic mycosis in North America, where it should be considered in immunocompromised hosts with systemic disease. *Es. africanus* causes the most common endemic mycosis in South Africa, primarily affecting HIV-infected persons (*7*); pulmonary and cutaneous disease are common, and the case-fatality rate is 50% (*8*). Invasive disease caused by *Es. pasteurianus* (previously *Emmonsia pasteuriana* [*2*,*9*]) has been reported from Italy, Spain, France, India, China, and South Africa (*3*). *Es. orientalis* was reported just once, from a man in China (*10*). Infection caused by another novel species, *Es. europaeus*, is known also from a single case from Germany (*11*).

Limited antifungal susceptibility testing of 2 *Es. canadensis* isolates found MICs elevated for fluconazole and low for itraconazole and amphotericin B. Dukik et al. (*12*) recently reported antifungal susceptibility results for 2 *Es. canadensis* isolates including UAMH 7172 (reported as CBS 139872) and UAMH 10370 (reported as CBS 1398723 The authors similarly found that MICs were elevated for fluconazole and low for newer triazoles and amphotericin B (*12*). These findings are consistent with the antifungal susceptibility patterns reported for 50 *Es. africanus* isolates (*7*). The anecdotal observation in our study that a patient (in case 2) remained fungemic with *Es. canadensis* 2 weeks after initiating fluconazole but had rapid clinical improvement with amphotericin B is consistent with these in vitro results. Pending the availability of further data, treatment of disease caused by *Emergomyces* spp. infection should follow clinical practice guidelines for the management of other dimorphic fungal infections in immunocompromised hosts (*13*). Specifically, treatment should include amphotericin B (lipid formulation 3–5 mg/kg or deoxycholate 0.7–1.0 mg/kg) for 1–2 weeks, followed by itraconazole (or other newer triazole) for at least 12 months, depending on immune reconstitution (*13*).

This report raises many questions about the pathogenesis, distribution, and habitat of *Es. canadensis*. As is the case for other dimorphic fungi, inhalational infection by *Emergomyces* spp. is presumed to occur, followed by extrapulmonary dissemination and disease in susceptible hosts (*3*). Although limited by small numbers and the lack of travel histories, these cases suggest that the geographic range of *Es. canadensis* likely involves central and western regions of North America. An ecologic niche has only been investigated for *Es. africanus*, which has been detected from various soil habitats and in air samples from Cape Town, South Africa (*14*,*15*). Further investigations are required to better understand the epidemiology and prevalence of disease caused by *Emergomyces* spp. in North America and globally.
